# Liver enzyme elevation in patients taking HAART compared with treatment naïve controls at Debre Berhan Referral Hospital: a comparative cross-sectional study, Northeast Ethiopia

**DOI:** 10.1186/s13104-019-4748-4

**Published:** 2019-10-30

**Authors:** Endalamaw Tesfa, Daniel Siefu, Yididya Belayneh, Zewdie Mekonnen

**Affiliations:** 1 Department of Biochemistry, College of Medicine and Health Sciences, Bahir Dar University, P.O. Box 79, Bahir Dar, Ethiopia; 2 Department of Biochemistry, College of Health Sciences, Addis Ababa University, Addis Ababa, Ethiopia

**Keywords:** HAART, HIV, Liver enzyme elevation, Hepatotoxicity, Debre Berhan

## Abstract

**Objective:**

HAART had significantly improved the quality of life of HIV patients. However, it results different adverse effects such as: hepatotoxicity, nephrotoxicity, lipodystrophy, anemia, diarrhea, psychiatric disorder and others. Therefore, this comparative cross sectional study was designed to investigate liver enzyme elevation in patients taking HAART compared with treatment naïve controls at Debre Berhan Referral Hospital.

**Result:**

A total of 152 individuals (76 cases and 76 controls) were included in this study. The mean ages of treatment and control groups were 37.37 and 36.38 respectively. The mean values of liver enzymes (ALT, AST and ALP), total bilirubin and direct bilirubin were significantly higher (p < 0.05) while, total protein and creatinine were significantly lower in patients taking HAART compared with treatment naïve controls. In this study, about 19 (25%) of clients in HAART treated groups and 7 (9.2%) of treatment naïve controls had showed liver enzyme changes. Moreover, 23.7% and 1.3% of the HAART treated groups developed mild and moderate liver enzyme elevation or hepatotoxicity, respectively. In this study, significant difference was observed in liver enzyme elevation between ART and pre-ART patients. As a result, regular clinical and laboratory monitoring of liver function will be necessary to prevent severe form of liver injury.

## Introduction

Highly active antiretroviral therapy (HAART) had significantly improved the quality of life of patients infected with human immunodeficiency virus (HIV) [1]. Despite, this positive effect, HAART give rise to adverse effects that lead to discontinuation of the treatment [2]. Adverse drug reactions might be asymptomatic or symptomatic. Symptomatic adverse effects may results in treatment failure, drug resistance and regimen change [1, 2]. Different drugs had distinct adverse effects. Patients getting stavudine (d4T) and protease inhibitor (PI) containing regimen were reported to develop lipodystrophy, insulin resistance and accelerated bone loss [3]. Patients receiving nevirapine containing regimen also developed liver toxicity and skin rash [4–6]. Similarly, Patients taking efavirenz containing regimen were also reported with psychiatric problem and night mares [7–9]. Zidovudine (AZT) also has been found to cause anemia and bone marrow suppression, and some ARV drugs were also reported to cause nephrotoxicity and lactic acidosis [1, 2].

Liver enzyme elevation is common problem that encounter in patients taking HAART. Antiretroviral (ARV) drugs damage the liver cells by direct toxicity of the drug or from its active metabolites. Duration of therapy and onset of liver disease provided a clue to the cause of liver injury. Liver injuries may be predictable or unpredictable. In case of predictable liver injury, toxicity might be related with the dose of the drug [10, 11]. From different studies the incidence of liver injury was different among different populations and different drug combinations [12]. In Ethiopia a study conducted at Debre Tabor hospital, the magnitude of abnormal liver enzymes in patients taking HAART and on pre-ART patients was 20.1% and 22% respectively. The study reported higher mean ALT level in patients taking HAART compared to pre-ART patients [13]. In another study conducted in Cameroon the incidence of hepatotoxicity was variable [14].

Although, many studies were conducted in different countries, they lack homogeneity on the magnitude and incidence of adverse effects of antiretroviral (ARV) drugs. This might be due to geographical and individual difference [15, 16]. In Ethiopia, there is few laboratory based researches done to understand the liver enzyme changes and the levels of elevation in patients taking ARV drug compared with treatment naïve controls. Therefore, this study was aimed to investigate liver enzyme elevation in patients taking HAART compared with treatment naïve controls at Debre Berhan Referral Hospital, Debre Berhan, Northeast Ethiopia.

## Main text

### Methods and materials

#### Study setting, population and sampling

Health institution based comparative cross sectional study was conducted from January to June 2015 at Debre Berhan Referral Hospital. The hospital is found at Debre Berhan town at a distance of 130 km from Addis Ababa, capital city of Ethiopia. Debre Berhan Referral Hospital provides different health service for about 2,045,992 people for North Shoa zone and people coming from neighboring regions like; Afar and Oromia [17]. ART service is one of the services that are provided by the hospital. During the study period, the hospital had 1527 HIV positive patients on HAART and 909 HIV positive patients on pre-ART.

Systematic sampling technique was applied to get a total sample size of 152 (76 people for each group). The sample size was estimated by double proportion formula through Epi-Info version 6 statistical packages by considering the risk of liver enzyme elevation on patients taking HAART was 2.5 times higher than the treatment naïve controls. The power of the study was assumed to be 80% at 95% confidence interval (CI) by allowing 5% margin of errors [18, 19].

#### Study variables and inclusion criteria

*Dependent variables* liver enzyme elevation (such as: ALT, AST, ALP, total protein, total and direct bilirubin). Liver enzyme elevation (Hepatotoxicity): operationally defined based on AIDS clinical trial group criteria or the Ethiopian ART guideline based on the serum ALT or AST values [2].

*Independent variables* demographic variables **(**age, sex, residence, education etc.).

*Inclusion criteria* volunteer respondents under HAART or pre-ART coming to the hospital during the data collection period and whose age ≥ 18 years were included in the study.

*Exclusion criteria* < 18 years old, clients who had known liver disease before HAART initiation, or individuals with hepatitis B or C virus positive were excluded

#### Ethical consideration

Ethical clearance was obtained from Addis Ababa University, College of Health Sciences ethical review committee. Permission was also requested and obtained from Debre Berhan Referral Hospital Manager and ART Focal person through a letter written from Addis Ababa University, College of Health Sciences. Data collection was started after we obtained written informed consent from each study participants.

#### Data collection and laboratory analysis

Demographic data were collected by using structured questionnaire through interview. 5 ml of whole blood was taken aseptically from pre-ART and ART patients by laboratory technologists. After formation of a clot, the serum was separated by centrifugation. Then the serum was screened for hepatitis B and C viruses to perform clinical chemistry parameters.

ALT, AST, ALP, total protein, total bilirubin, direct bilirubin, urea and creatinine tests were analyzed via Spain *A25* Biosystem automated chemistry machine analyzer as per the manufacture instruction. The machine is controlled through software installed on dedicated PC that constantly informs the user about the status and progress of analyses (*A25* Biosystem Spain, 2007). Fluorescence-activated cell sorter (FACS) (Becton–Dickinson, USA) was used to determine the absolute CD4+ T cells count. Hemoglobin was measured through hematology analyzer MindrayBC320 (Mindray Biomedical electronic Corporation, China). Before the laboratory analysis the chemistry machine analyzer was calibrated and validated. In addition internal quality control was performed to maintain the quality of data.

#### Statistical analysis

All data analyses were performed using SPSS version 21 software. Data were presented as means and calculations carried out using the Student’s independent t-test. Binary and multiple logistic regression models were utilized to assess associations of predictor variables and laboratory outcomes. p-value < 0.05 was taken as a cut point at 95% CI.

### Results

#### Demographic characteristics

A total of 152 individuals (76 HAART treated and 76 treatment naïve controls) were included in this study. The mean age of HAART treated groups and treatment naïve controls were 37.37 and 36.38 respectively. Most of the study participants were females and urban dwellers. More than half of the study participants had attended primary education and above (Table 1).

**Table 1:** Demographic characteristics of study participants (HAART treated group and treatment naïve controls) at Debre Berhan Referral Hospital Northeast Ethiopia

Variables	Frequency of ART group (n (%))	Frequency of pre-ART group (n (%))
Age		
Age in mean	37.37	36.38
Sex		
Male	31 (40.8)	30 (39.5)
Female	45 (59.2)	46 (60.5)
Marital status		
Single	12 (15.8)	16 (21.1)
Married	34 (44.7)	33 (43.4)
Divorced	13 (17.1)	19 (25)
Widowed	17 (22.4)	8 (10.5)
Residence		
Urban	65 (85.5)	57 (75)
Rural	11 (14.5)	19 (25)
Educational status		
No formal education	23 (30.3)	36 (47.4)
Primary education	28 (36.8)	19 (25)
Secondary and above	25 (32.9)	21 (27.6)

#### Laboratory results

Table 2 summarized the mean levels of liver enzymes between HAART treated group and treatment naive controls and 95% CI for mean. HIV positive HAART treated group had significantly higher mean values of ALT, AST, ALP, total bilirubin and direct bilirubin when compared with treatment naïve controls. On the other hand, the mean values of total protein and creatinine level were significantly lower in patients taking HAART compared with treatment naïve controls. Urea, hemoglobin and CD4-T-cell count didn’t show any significant difference between the two groups.

**Table 2:** Independent T-test of mean and 95% CI for mean level of liver enzymes of study participant at Debre Behan Referral Hospital, Northeast Ethiopia

Parameter	Study group	Number	Mean	95% CI for mean	p-value
ALT (U/L)	ART group	76	35.67	32.18–39.16	0.000*
	Pre-ART group	76	23.97	21.38-26.57	
AST (U/L)	ART group	76	36.05	32.88–39.23	0.000*
	Pre-ART group	76	28.00	25.52–30.49	
ALP (U/L)	ART group	76	117.14	97.99–136.30	0.022*
	Pre-ART group	76	89.45	75.27–103.64	
Total protein (g/dL)	ART group	76	7.09	6.89–7.30	0.007*
	Pre-ART group	76	7.46	7.29–7.64	
Total bilirubin (mg/dL)	ART group	76	0.787	0.761–0.813	0.000*
	Pre-ART group	76	0.660	0.649–0.673	
Direct bilirubin (mg/dL)	ART group	76	0.275	0.227–0.324	0.007*
	Pre-ART group	76	0.188	0.149–0.228	
Urea (mg/dL)	ART group	76	19.945	18.414–21.477	0.875
	Pre-ART group	76	19.750	17.824–21.678	
Creatinine (mg/dL)	ART group	76	0.588	0.562–0.614	0.016*
	Pre-ART group	76	0.641	0.607–0.675	
Hemoglobin (g/dL)	Treatment group	76	15.44	14.98–15.91	0.245
	Pre-ART group	76	15.02	14.46–15.58	
CD4 count (cells/µL)	ART group	76	428.14	367.75–488.53	0.362
	Pre-ART group	76	390.97	336.93–445.01	

* Statistically significant

#### Liver enzyme elevation

In the current study, about 19 (25%) of the study participants of the HAART treated group and 7 (9.2%) of treatment naïve controls showed liver enzyme elevation. There was significant difference (p < 0.05) in the levels of liver enzyme elevation between the two groups with the odds of 3.095 (1.213, 7.898). In HAART treated groups 18 (23.7%) patients were developed mild liver enzyme elevation and also 1 (1.3%) patient developed moderate liver enzyme elevation.

#### Association of independent variables with utilization of ARV therapy

Nine independent variables were analyzed in multiple logistic regressions by considering the dependent variable ALT/AST level to understand their association. Out of nine independent variables that were analyzed in multiple logistic regressions, BMI was found statistically significant. Patient’s whose body mass index (BMI) existed between 18.6 and 24.9 were 3 times more likely to developing ALT or AST elevation (Table 3).

**Table 3:** Summarizing the association of independent variable with ALT/AST level via logistic regression analysis in patients taking HAART compared with treatment naïve controls at Debre Berhan Referral Hospital, Northeast Ethiopia

Variables	ALT/AST Level		COR	Sig	AOR
	Normal	Elevated			
Age					
≤ 35 years	63 (41.4)	14 (9.2)	1	1	1
> 35 years	63 (41.4)	12 (7.9)	1.167 (0.500–2.210)	0.721	1.152 (0.438–3.031)
Sex					
Male	52 (34.2)	9 (5.9)	1	1	1
Female	74 (48.7)	17 (11.2)	0.753 (0.312–1.821)	0.529	1.255 (0.427–3.690)
Marital status					
Single	24 (15.8)	4 (2.6)	1	1	1
Married	54 (35.5)	15 (9.9)	0.600 (0.180–1.998)	0.405	0.510 (0.137–1.908)
Divorced	28 (18.4)	3 (2)	1.556 (0.316–7.652)	0.587	1.947 (0.311–12.184)
Widowed	20 (13.2)	4 (2.6)	0.433 (0.140–1.338)	0.813	1.019 (0.178–5.826)
Residence					
Urban	98 (64.5)	23 (15.1)	1	1	1
Rural	28 (18.4)	3 (2)	2.190 (0.613–7.834)	0.228	0.304 (0.072–1.280)
Educational status					
No formal education	23 (15.1)	9 (5.9)	0.249 (0.069–0.900)	0.034	0.130 (0.029–0.592)
Primary education	62 (40.8)	13 (8.6)	0.465 (0.142–1.527)	0.207	0.338 (0.092–1.242)
Secondary and above	41 (27)	4 (2.6)	1	1	1
Clinical stage					
Stage I	45 (29.6)	8 (5.3)	1	1	1
Stage II	30 (19.7)	6 (3.9)	0.889 (0.280–2.821)	0.842	1.491 (0.364–6.108)
Stage III	42 (27.6)	11 (7.2)	0.679(0.249–1.851)	0.449	0.607 (0.193–1.908)
Stage IV	9 (5.9)	1 (0.7)	1.600 (0.178–14.42)	0.675	1.635 (0.151–17.723)
Body mass index					
< 18.5	22 (14.5)	9 (5.9)	1	1	1
18.6–24.9	88 (57.9)	15 (9.9)	2.400 (0.929–6.201)	0.071^a^	3.387 (1.119–10.265)
≥ 25	16 (10.5)	2 (1.3)	3.273 (0.621–17.247)	0.162	3.333 (0.536–20.733)
CPT-prophylaxis					
Yes	61 (40.1)	18 (11.8)	0.417 (0.169–1.029)	0.058	0.280 (0.093–0.842)
No	65 (42.8)	8 (5.3)	1	1	1
Regimens					
d4T-3TC-NVP	8 (10.5)	2 (2.6)	1.455 (0.212–9.984)	0.703	2.710 (0.100–73.157)
d4T-3TC-EFV	2 (2.6)	2 (2.6)	0.364 (0.038–3.518)	0.382	0.308 (0.003–33.145)
ZDV-3TC-NVP	16 (21.1)	3 (3.9)	1.939 (0.361–10.430)	0.440	5.898 (0.287–121.288)
ZDV-3TC-EFV	11 (14.5)	3 (3.9)	1.212 (0.216–6.800)	0.827	2.403 (0.133–43.467)
TDF-3TC-NVP	9 (11.8)	5 (6.6)	0.655 (0.134–3.186)	0.600	0.519 (0.028-9.727)
TDF-3TC-EFV	11 (14.5)	4 (5.3)	1	1	1

*COR* crude odd ratio, *AOR* adjusted odds ratio, *d4T* stavudine, *3TC* lamivudine, *NVP* nevirapine, *EFV* efavirenz, *ZDV* zidovudine, *TDF* tenofovir

^a^Multiple logistic regression statically significant, ALT normal value for male ULN ≤ 40 U/L, for female ALT ≤ 35 U/L and AST value for male ≤ 35 U/L and AST value for female ≤ 31 U/L

### Discussion

This study tried to evaluate the effect of HAART on liver enzyme elevation in patients taking HAART and treatment naive controls. Liver enzyme elevation might be occurring through different causes like; drugs, toxins, HBV, BCV, HIV, endogenous metabolite and alcohol [15, 20–23]. Different ARV drugs have been reported to affect liver enzyme activity [24, 25]. The level of liver cell injury is usually assessed by measuring the plasma concentration of transaminase enzymes [26]. It is obvious that liver is the site of synthesis of thousands of enzyme. When there is any insult on liver, these enzymes were released into the plasma resulted in increased in concentration [27]. ALT and AST are the most sensitive indicators of liver cell injury and used for the diagnosis of acute hepatocellular disease [28].

There were significant differences (p < 0.05) in the mean values of ALT and AST between the two groups. This significant difference was explained due to the adverse effects of ARV drugs. ALT is a sensitive marker, primarily secreted in liver cells, while AST is produced in liver and other tissues like; heart, skeletal muscle, kidneys, brain, pancreas, lungs, leukocytes, and erythrocytes [27]. These enzymes are released into the plasma in greater amounts when there is an injury to the liver cell membrane resulting in increased permeability. Different research out puts indicated that patients taking ARV drugs had shown elevated levels of ALT and AST [28–30]. In this study, the mean value of ALP was significantly increased in HAART treated groups compared with treatment naïve controls. It is produced in the bile duct or near to the bile canalicular membrane of hepatocytes, intestine, kidney, placenta and bone [27]. The observed significant difference in ALP might be due to the side effect of ARV drugs that leads to increased production of the liver ALP isoenzyme [28].

In this study, the mean value of total protein was significantly (p < 0.05) reduced in HAART treated groups compared to treatment naïve controls. The drop in protein synthesis in patients taking HAART might be due to drug induced hepatic damage that results in reduced synthesis of different plasma proteins [2, 31]. The mean levels of total and direct bilirubin were significantly (p < 0.05) increased in patients taking HAART compared with treatment naïve controls. This could be due to the effect of ARV drugs on liver that resulted in decreased processing of bilirubin [28]. Moreover, significantly lower mean level of creatinine was observed in HAART treated groups than treatment naïve controls, while the mean values of urea and hemoglobin were not statistically significant (p > 0.05) between the two groups.

In the current study, the overall liver enzyme elevation was 25% in patients taking HAART groups and 9.2% in treatment naïve controls. In this study severe form of liver enzyme elevation was not observed. However, different studies reported that ARV drugs resulted in different degrees of liver enzyme elevation or hepatotoxicity (such as; mild, moderate and severe). This might be due to a number of factors such as; co-infection with hepatitis virus, the habit of alcohol ingestion, the regime of the drug, the duration of treatment, presence or absence of comorbid conditions, geographic condition and genetic polymorphisms contributed for these differences in the level of liver injury among various studies [4, 6, 21, 32–37].

In this study, multiple logistic regression analysis of socio-demographic and clinical variables didn’t show any statistical significant (p > 0.05) association on liver enzyme elevation based on (ALT/AST) values except BMI. Patients who had BMI between 18.5 and 24.9 were 3 times more likely to develop liver enzyme elevation (ALT/AST) than their counter parts. This might be due to the fact that BMI correlated with the drug metabolism properties of the patient [25, 33].

## Conclusion

In this study, liver enzymes (ALT, AST, ALP, total protein, creatinine, total and direct bilirubin) were significantly elevated in patients taking HAART compared with treatment naive controls. About 25% of patients taking HAART developed mild and moderate liver enzyme elevation but 9.2% of treatment naïve controls developed mild liver enzyme alteration. As a result, regular clinical and laboratory monitoring will be necessary to prevent severe form of liver toxicity.

## Limitation of the study

This comparative cross sectional study was becomes strong if the study design becoming a cohort. The study conducted on 152 study participants these number somewhat small to give an inference on the general population about the effects of HAART on liver enzymes in patients taking HAART. Due to financial issues this study didn’t include all relevant liver enzymes that are employed for the diagnosis of liver injury (for instance γ-GGT).

## Data Availability

All relevant data are included within the manuscript. If it is necessary it is possible to contact the corresponding author to get additional material.

## Abbreviations

ALT: alanine aminotransferase
AST: aspartate aminotransferase
ALP: alkaline phosphatase
Hb: hemoglobin
CD4+: cluster of differentiation
U/L: unit per liter
mg/dL: milligram per deciliter
g/dL: gram per deciliter
cells/µL: cells per micro liter
HAART: highly active antiretroviral therapy
ART: antiretroviral therapy
ULN: upper limit normal
WHO: world health organization
HIV: human immune deficiency virus
AIDS: acquired immuno deficiency syndrome
HBV: hepatitis B virus
HCV: hepatitis C virus
ANOVA: analysis of variance
COR: crude odds ratio
AOR: adjusted odds ratio
CPT: cotrimoxazole preventive therapy
BMI: body mass index
CI: confidence interval
SPSS: statistical package for social sciences
PI: protease inhibitor
d4T: stavudine
DNA: deoxyribonucleic acid
FHAPCO: Federal HIV/AIDS Prevention and Control Office
rpm: revolution per minutes
ECSA: Ethiopian central statistics agency
FACS: fluorescence-activated cell sorter
